# Combination of clozapine, cariprazine and fluoxetine in treatment-resistant schizophrenia patient with prominent negative symptoms: A Case report

**DOI:** 10.1192/j.eurpsy.2022.2017

**Published:** 2022-09-01

**Authors:** I. Everte, A. Pamše-Romāne, M. Taube

**Affiliations:** 1 Strenci Psychoneurological hospital, 4th Department Of Psychiatry, Strenci, Latvia; 2 Riga Centre of Psychiatry and narcology, Department Of Depression And Crisis Situations (20th), Riga, Latvia

**Keywords:** A case report, psychiatry, schizophrénia, treatment-resistant schizophrenia

## Abstract

**Introduction:**

Despite pharmacological advances in the treatment of schizophrenia, significant number of patients are still treatment-resistant. Clozapine is recommended as first-line treatment for treatment-resistant schizophrenia (TRS) in guidelines. Despite the greater efficacy of clozapine over other antipsychotics in the management of TRS, a significant number of patients fail to attain adequate response or develop adverse effects, and more interventions are needed.

**Objectives:**

To describe a clinical case of treatment-resistant schizophrenia patient with prominent negative symptoms treated with combination of clozapine, cariprazine and fluoxetine, and to review the literature.

**Methods:**

Clinical case presentation through review of the clinical file and non-systematic review on PubMed and ResearchGate.

**Results:**

A 41 year old female patient presented to inpatient clinic with low mood, occasional commanding and commenting verbal hallucinations, occasional suicidal thoughts, blunted affect, anhedonia, asociality, she was apathetic, lacked motivation to get up from bed, had night’s sleep disturbance. Patient was diagnosed with Schizophrenia in 2009, she has been hospitalized in Psychiatric wards for 16 times. She has received treatment with combinations of several antipsychotic drugs and antidepressants, had side-effects and have not reached full remission. During treatment with clozapine (up to 175mg per day) in combination with cariprazine (up to 4.5mg per day) and fluoxetine (up to 20mg per day), gradually negative symptoms decreased, patient became more active, showed interest in daily and rehabilitation activities, night’s sleep improved.

**Conclusions:**

Patient with treatment-resistant schizophrenia benefited from combination of clozapine, cariprazine and fluoxetine. Further research is necessary on treatment combination strategies for TRS.

**Disclosure:**

No significant relationships.

